# Relationship key factor of inflammation and the development of complications in the late period of myocardial infarction in patients with visceral obesity

**DOI:** 10.1186/s12872-017-0473-x

**Published:** 2017-01-19

**Authors:** Olga Gruzdeva, Evgenya Uchasova, Yulia Dyleva, Olga Akbasheva, Vera Matveeva, Victoria Karetnikova, Alexander Kokov, Olga Barbarash

**Affiliations:** 1 Federal State Budgetary Institution “Research Institute for Complex Issues of Cardiovascular Disease”, Kemerovo, Russia; 2 Federal State Budget Educational Institution of Higher Professional Education “Siberian State Medical University” of the Ministry of Healthcare of the Russian Federation, Tomsk, Russia

## Abstract

**Background:**

Cytokines play an significant role in regulating non-specific inflammatory response involved in many pathological processes. The current study tested the hypothesis that myocardial infarction in patients with obesity can lead to increased production of proinflammatory cytokines and unfavorable course of the pathological process.

**Methods:**

The study recruited 232 male patients with ST-elevated myocardial infarction. The mean age of the patients was 58.7 (52.2-69.9) years. All the patients were assigned to two groups according to the computed tomography findings: 1 (*n* = 160) patients with visceral obesity (VO), and 2 (*n* = 72) patients without VO. Interleukins were measured in blood serum on days 1 and 12 after MI.

**Results:**

All patients with MI demonstrated elevated levels of proinflammatory markers and reduced anti-inflammatory markers in the in-hospital period. The results suggested that among all studied inflammatory markers IL-6 (OR 1.9; 95% CI (1.6–2.8) and CRP (OR 1.3; 95% CI (1.1–1.8) were closely related to VO. One year after MI adverse cardiovascular outcome frequently occurred in patients with VO. There were two cardiac deaths (3.1%), 6 cases (9.3%) of recurrent MI, 19 cases (29.6%) of repeated hospitalizations for unstable angina, whereas only 2 patients without VO (6.6%) were hospitalized for unstable angina. The results of the logistic regression analysis demonstrated that IL-6, IL-12, and IL-10 had the highest predictive value for occurrence of adverse cardiovascular events in patients with VO.

**Conclusion:**

Cytokine profile in MI patients with VO is characterized by an imbalance caused by elevated pro-inflammatory interleukins and decreased anti-inflammatory interleukins. Obesity in patients was associated with a marked increase in IL-6 and CRP levels.

## Background

Cytokines play an significant role in regulating non-specific inflammatory response involved in many pathological processes [1]. Pro-inflammatory (TNF-α, IL-1β, IL-6, IL-8 and IL-12) and anti-inflammatory (IL-10) cytokines defines adaptive course of inflammation. An imbalance in the can lead to chronic inflammation. Chronic inflammation is a key factor in the initiation and progression of atherosclerosis that ultimately results in the destabilization of atherosclerotic plaques, coronary artery thrombosis, myocardial infarction (MI) [1]. Obesity-induced adipose tissue inflammation is considered to be an independent risk factor for cardiovascular disease (CVD), which is the leading cause of death and disability among working-age people in developed countries [2]. Cytokines are produced mainly by immune system cells and adipocytes [3]. The expression of the anti-inflammatory cytokines is stimulated in adipose tissue of healthy subjects, while large quantities of pro-inflammatory cytokine are secreted in patients with CVD [4]. The current study tested the hypothesis that myocardial infarction in patients with obesity can lead to increased production of proinflammatory cytokines and unfavorable course of the pathological process.

### Purpose

To study the relationships between key inflammatory factors and complications in the late post myocardial infarction period in patients with visceral obesity.

## Methods

The study recruited 232 male patients with MI. Acute MI was diagnosed according to the 2007 Russian National Cardiology Society guidelines and ESC/ACCF/AHA/WHF based on clinical (presence of typical pain lasting longer than 15 min), electrocardiographic (ST-segment elevation of 0.1 mW in two or more contiguous leads), echocardiographic and biochemical signs (elevated creatine phosphokinase, creatine phosphokinase-MB, troponin T levels(>0,1 ng/ml).

The exclusion criteria were as follows: age <50 or >80 years, the presence of T2DM, and a prior history of pronounced renal failure (glomerular filtration rate <30 mL/min). Also excluded from the study were excluded HIV-infected patients, and cancer patients with known pathology [5].

All patients provided written informed consent prior to their participation in the study.

The mean age of the patients was 58.7 (52.2–69.9) years.

All the patients underwent multi-slice computed tomography (CT) using a Lightspeed VCT 64 (General Electric, Fairfield, CT, USA) to measure abdominal adipose tissue. Visceral adipose tissue (VAT) and subcutaneous adipose tissue (SAT) areas as well as the ratio of VAT to SAT were measured. Two diagnostic criteria (the proposed method L. Sjoestrom) were used to confirm visceral obesity (VO): VAT area >130 cm^2^ and the ratio of VAT to SAT ≥0.4 [6].

All the patients were assigned to two groups according to the CT findings: Group 1 (*n* = 160) patients with VO, and Group 2 (*n* = 72) patients without VO.

The clinical and demographic data are shown in Table 1. All the patients underwent primary percutaneous coronary intervention of the infarct-related artery as a reperfusion therapy. The control group included 30 males without diagnosed CVD and comparable in age and sex with the patients included in the study (aged 58.42 (52.2–61.1) years). The CT findings demonstrated that none of the control subjects suffered from VO (VAT area was 110.0 [104.0–128.0] cm^2^ and the VAT/SAT ratio 0.35 [0.2–0.39]).

**Table 1:** Baseline clinical characteristics of patients

Variable	Patients with visceral obesity, *n* = 160	Patients without visceral obesity, *n* = 72	*p*
Men	58 (54;69)	57 (50;67)	0.92
Arterial hypertension, *n* (%)	160 (100.0)	60 (83.3)	0.05
Current smoking	65 (40.6)	39 (54.2)	0.46
Family history of IHD	105 (65.6)	24 (33.3)	0.04
Family history of T2DM	35 (21.9)	8 (11.1)	0.04
Features history			
Angina prior to myocardial infarction	85 (53.1)	48 (66.6)	0.69
Previous myocardial infarction	31 (19.3)	13 (18.1)	0.051
History of heart failure	16 (10)	8 (11.1)	0.81
History of cerebrovascular accident/transient ischemic attack	0	3 (4.2)	0.98
Myocardial infarction			
Q-wave myocardial infarction	128 (80)	58 (80.5)	0.61
Non-Q-wave myocardial infarction	33 (20.3)	15 (20.8)	0.62
Localization of myocardial infarction			
- posterior	105 (65.6)	38 (54.2)	0.61
- posterior with extension to the right			
ventricle	18 (12.3)	10 (13.9)	0.72
- anterior	30 (18.8)	20 (27.8)	0.59
- inferio- posterio-lateral	8 (5)	6 (8.3)	0.79
Acute heart failure (Killip classification)			
I	110 (68,8)	44 (61.1)	0.52
II	31 (19.4)	17 (23.6)	0.64
III	15 (9,4)	10 (13.8)	0.72
IV	3 (1.9)	0	0.95
- Rhythm disturbance	38 (23.8)	20 (27.8)	0.78
Early post-infarction angina	30 (18.8)	16 (22.2)	0.71
Recurrent myocardial infarction	10 (6.3)	3 (4.2)	0.91
Creatine phosphokinase, U/L	339.2 (203.1;699.4)	245 (110.7;523.1)	0.03
Max creatine phosphokinase-MB, U/L	81 (33;179)	66 (35;142)	0.03
Troponin T, ng/ml	1.01 (0.82;3.1)	0.69 (0.17;1.2)	0.01
Left ventricular ejection fraction, %	50 (40;57)	52 (42;53)	0.60
Number of diseased coronary arteries			
Stenosis of a vessels	28 (17.5)	16 (22.2)	0.72
Stenosis of 2 vessels	15 (9.4)	26 (36.1)	0.03
Stenosis of 3 or more vessels	122 (76.3)	14 (43.1)	0.03
Treatment strategy/group of drugs			
Stenting of the infarct-related artery	160 (100)	72 (100)	0,81
Systemic thrombolytic therapy	13 (8.1)	10 (13.8)	0.73
β-blockers	140 (87.5)	72 (100)	0,82
Angiotensin-converting enzyme	145 (90.6)	68 (42.5)	0.59
Calcium channel blocker	120 (78.1)	62 (38.8)	0.97
Diuretics	53 (33.1)	26 (36.1)	0.81
Nitrates	26 (15.6)	15 (20.8)	0.80
Aspirin	160 (100)	71 (98.6)	0.91
Heparin	160 (100)	72 (100)	0.98
Clopidogrel	148 (92.5)	62 (95.8)	0.81
Statins	160 (100.0)	72 (100.0)	0.93

*P*-value for differences between groups (*P* < 0.05). Data are expressed as number (percentage)

Abbreviations: *HIS* ischemic heart disease; T2DM, type 2 diabetes mellitus

### Blood sampling and biochemical assays

The serum of each patient was separated from blood by centrifugation at 3 000 × *g* for 20 min and stored at −70 °C. Proinflammatory markers were measured in blood serum on days 1 and 12 after MI. Serum concentrations of interleukins (IL-1β, IL-6, IL-8, IL-10 IL-12 and TNF-α,) were determined with ELISA using the Monobind ELISA test systems (USA). C-reactive protein (CRP) levels were measured using a standard Thermo Fisher Scientific test system (Thermo Fisher Scientific Oy, Vantaa, Finland) in a Konelab 30i biochemistry analyzer (Thermo Fisher Scientific Oy).

### Statistical analysis

Statistical analysis was performed using Statistica 6.1 (InstallShield Software Corp., Chicago, IL, USA) and SPSS 17.0 for Windows (SPSS Inc., Chicago, IL, USA). The Kolmogorov–Smirnov test was used to assess the distribution of two data sets. Results are presented as median (Me) and 25 and 75% quartiles Me (Q1;Q3). The statistical analysis was performed using the non-parametric Mann–Whitney test for skewed distributions. Stepwise logistic regression and a receiver operating characteristic (ROC) curve with the area under the curve (AUC) measurement were used to determine the most informative VO parameters, the hazard ratio (HR) and the confidence interval (95%). *P* values <0.05 were considered statistically significant.

## Results

All patients with MI demonstrated elevated levels of proinflammatory markers (TNF-α, IL1β, IL6, IL8, IL12, CRP) and reduced anti-inflammatory (IL-10) markers in the in-hospital period. However, these changes were more pronounced during the whole follow-up period in the presence of VO (Table 2).

**Table 2:** Markers of inflammation in patients with myocardial infarction with and without visceral obesity, Me (Q1;Q3)

Variable	Control group, *n* = 30	Patients with visceral obesity, *n* = 160		Patients without visceral obesity, *n* = 72	
		1st day	12st day	1st day	12st day
	1	2	3	4	5
TNF-α, pg/ml	1.2 (0.9;1.4)	1.4 (1.0;1.7) p_1_-_2_ = 0.01	1.9 (1.4;2.0) p_1–3_ = 0.01 p_2–3_ = 0.02	1.2 (0.7;1.6) p_2–4_ = 0.002	1.0 (0.8;2.1) p_3–5_ = 0.003
IL -1β, pg/ml	2.2 (2,1;4,3)	5.2 (2.6;6.2) p_1–2_ = 0,001	4.9 (2.7;6.6) p_1–3_ = 0.002	3.3 (2.0;4.4) p_2–4_ = 0.003	2,4 (1.1;5.4) p_3–5_ = 0.002
IL −6, pg/ml	2.55 (2,1;3,3)	17.5 (11.7; 25.1) p_1–2_ = 0.001	9.5 (3.2; 4.3) p_1–3_ = 0.01 p_2–3_ = 0.02	12.0 (6.9;18.7) p_1–4_ = 0.006 p_2–4_ = 0.004	6.1 (2.5;14.1) p_1–5_ = 0.002 p_3–5_ = 0.001 p_4–5_ = 0.01
IL −8, pg/ml	2.4 (2.1;4.1)	58.0 (29.9;69.5) p_1–2_ = 0.001	48,3 (40.4;64.4) p_1–3_ = 0.00 p_2–3_ = 0.01	45.5 (27.4;54.7) p_1–4_ = 0.005 p_2–4_ = 0.004	43,6 (35,3;52.2) p_1–5_ = 0.003 p_3–5_ = 0.004
IL −12, pg/ml	60.4 (47.2;88.6)	128.7 (66.4;182.0) p_1–2_ = 0.001	98.4 (86.7;261.2) p_1–3_ = 0.005 p_2–3_ = 0.02	100.1 (48.0;151.7) p_1–4_ = 0.007 p_2–4_ = 0.03	55.3 (44.0;101.3) p_3–5_ = 0.02 p_4–5_ = 0.01
CRP, mg/l	1.0 (0.8;1.5)	23.2 (12.1;54.1) p_1–2_ = 0.008	11.3 (5.0;21.6) p_1–3_ = 0.00 p_2–3_ = 0.01	20.2 (12.8;35.0) p_1–4_ = 0.003 p_2–4_ = 0.001	7.7 (4.7;15.0) p_1–5_ = 0.005 p_3–5_ = 0.004 p_4–5_ = 0.01
IL −10, pg/ml	8.9 (7.4;10.2)	1.9 (0.7;2.5) p_1–2_ = 0.00	3.8 (1.1;4.5) p_1–3_ = 0.00 p_2–3_ = 0.01	5.6 (3.2;6.2) p_1–4_ = 0.00 _p2–4_ = 0.02	7.8 (6.8;9.7) p_1–5_ = 0.00 p_3–5_ = 0.01 p_4–5_ = 0.01

Data in the table are presented as median (Me) and 25% and 75% quartiles (Q1;Q3)

*P*-value for differences between groups (*P* < 0.05

TNF-α and IL-1β are the first line cytokines that are produced in response to inflammatory stimuli. The production and secretion of cytokines may not be predominantly caused by myocardial ischemia-reperfusion injury, but also by macrophages infiltrating adipose tissue, and adipocytes. Thus, statistically significant increase in the levels of TNF-α and IL-1β was found in patients with VO on days 1 and 12 after MI (Table 2). TNF-α and IL-1β levels in patients with VO on day 1 after MI was 1.2- and 1.6-fold higher compared to patients without excess VO. On day 12, there was a 1.4-fold increase in TNF-α levels compared to the values obtained on day 1. However, IL-1β levels did not change significantly. Patients without VO reported no significant differences in the levels of cytokines compared to the levels in the control group during the study.

On day 1, IL-12 levels were relatively elevated in patients with VO (a 2.1-fold increase), and without VO (a 1.6-fold increase) compared to the levels in the control group. Importantly, the concentration of IL-12 was 1.3-fold higher in patients with VO than in those without VO. On day 12, IL-12 levels decreased in both groups, but it did not achieve the control group levels in patients with VO.

More significant changes were observed in IL-6 levels. Patients with VO on day 1 after MI reported a 6.9- and 1.45-fold increase in IL-6 levels compared to the control group and patients without VO. Despite the reduction in the concentration of the cytokine on day 12 in both groups, the values in healthy subjects were not achieved. Importantly, there was a 1.6-fold increase in IL-6 levels in patients with VO compared to patients without VO.

The production of IL-8, a chemokine produced by macrophages and neutrophils, and CRP, an acute phase protein, modulating the immune responses, are activated by proinflammatory cytokines (TNF-α, IL-1β, IL-6 and IL-12). Apparently, this amplifying effect of cytokines has led to the most intense changes in the concentrations of IL-8 and CRP compared to other pro-inflammatory factors. Thus, patients with VO on days 1 and 12 demonstrated a 24.2- and 20.1-fold increase in IL-8 levels compared with healthy subjects. Moreover, this increase was statistically significant compared to patients without VO (Table 2). On day 1 after MI, patients with VO reported a 23.2-fold increase in CRP concentrations compared with the levels in the control group. On day 12, a 2-fold decrease was found in patients with VO, but the levels of the control group were not achieved. The levels of CRP in patients with VO increased 10-fold the levels in the control group. On day 12, patients without VO demonstrated a 7.7-fold increase compared to the levels in the control group.

IL-10 is the central anti-inflammatory cytokines, blocking synthesis of pro-inflammatory cytokines. On day 1 after MI, patients with VO demonstrated a pronounced deficit of IL-10. MI patients with VO reported a 78% reduction in IL-10 levels compared to the control group, whereas patients without VO had a 37% reduction. On day 12, IL-10 increased 2- and 1.4-fold in both groups, respectively. However, a 2-fold decrease in IL-10 levels remained in patients with VO compared with patients without VO.

IL-8/IL-10 ratio was calculated to assess the imbalance of pro- and anti-inflammatory cytokines. On days 1 and 12, the ratio in patients with VO were 30.5 and 12.7, suggesting a 3.8- and 2.3-fold increase compared to the values in patients without VO (8.13 and 5.6, respectively). Increased IL-8/IL-10 ratio was associated with elevated IL-8 levels and decreased IL-10 levels. Thus, the imbalance of pro- and anti-inflammatory cytokines was more pronounced in the presence of visceral obesity.

The logistic regression analysis was performed to identify the most informative variables. The results suggested that among all studied inflammatory markers IL-6 IL-6 (OR 1.9; 95% CI (1.6-2.8) AUC = 0.80, *p* = 0.01) and CRP (OR 1.3; 95% CI (1.1-1.8) AUC = 0.77, p = 0.02) were closely related to visceral obesity.

**Fig. 1:**
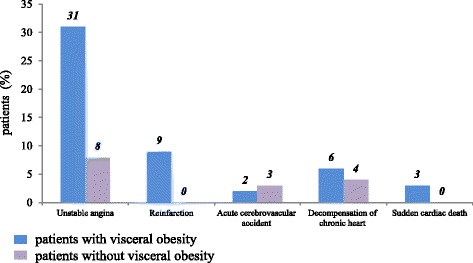
Basic cardiovascular events within 1 year after myocardial infarction in patients with and without visceral obesity, n (%). The differences between study groups are statistically significant (*P* < 0.05)

One year after MI adverse cardiovascular outcome frequently occurred in patients with VO (Fig. 1). There were two cardiac deaths (3.1%), 6 cases (9.3%) of recurrent MI, 19 cases (29.6%) of repeated hospitalizations for unstable angina, whereas only 2 patients without VO (6.6%) were hospitalized for unstable angina. Importantly, there were no cases of cardiac death and recurrent MI in the group of patients without VO.

The results of the logistic regression analysis demonstrated that IL-6 (OR 1.9; 95% CI (1.5-2.1), AUC = 0.84, *p* = 0.02), IL-12 (OR 1.3; 95% CI (1.1-2.0) AUC = 0.75, *p* = 0.032, and IL-10 (OR 0.8; 95% CI (0.5-0.9) AUC = 0.75, *p* = 0.04) had the highest predictive value for occurrence of adverse cardiovascular events in patients with VO. Elevated levels of IL-6 and IL-12 were associated with a 1.9- and 1.3-fold increased risk for developing cardiovascular complications, respectively. Moreover, reduced levels of anti-inflammatory cytokine, IL-10, was accompanied by a 20% increased risk, compared to patients without VO.

## Discussion

It should be noted that visceral adipose tissue is considered to be one of the most harmful due to its positive association with the development of cardiovascular disease [1]. Excess adiposity is characterized as a chronic state of low-grade inflammation or so-called metabolic inflammation [2]. Adipose tissue-resident immune cells (primarily lymphocytes and macrophages) and hypertrophied adipocytes both contribute to increased circulating levels of proinflammatory cytokines [7]. White adipose tissue (WAT) is the key site mediating systemic inflammation since it is virtually around all organs and tissues, and occupies a large area in obese people [8].

The results of our study suggest that the presence of VO in MI patients is associated with elevated levels of pro-inflammatory factors (TNF-α, IL-1β, IL-6, IL-8, IL-12, CRP), which are mainly produced by macrophages, localized around hypertrophied adipocytes, accumulating in both the subcutaneous and visceral expanding fat depots, even though macrophage infiltration appears to be more prominent in the latter [9].

Along with an increase in the number of adipose tissue macrophages (ATM) for obesity undergo phenotypic changes. In obesity the white oil contains mostly proinflammatory macrophage M1 (40%), while in normal anti-inflammatory predominant population of M2 macrophages. [4]. Activated M1 ATMs are a prominent source of proinflammatory cytokines such as TNF-α and IL-6, IL-8 and may be regarded as effectors of a coordinated inflammatory response. Thus, ATM polarization into M1 to M2, being a more pronounced in visceral obesity, might provide new insights into worsening inflammatory response and reducing anti-inflammatory resistance.

Another important issue that may activate proinflammatory potential in obesity is an increase of expression of Toll-like receptors (TLR2, TLR4) on the membranes of various cell types, such as immune and resident non-immune cells, including adipocytes in VAT [10]. Activation of Toll-like receptors causes the release of transcriptional factors that activate genes responsible for the synthesis of various bioactive molecules, including proinflammatory cytokines (TNF-α, IL-1β, IL-6, IL-8, IL-12) [11].

Ligands can be derived not only from pathogens, but they also can be endogenous, such as heat shock proteins (HSPs) [12], extracellular matrix degradation products (hyaluronan, biglycan, fragments of heparan sulphate) [13], saturated fatty acids (SFAs) of exogenous and endogenous origin are recognized by TLR2 or TLR4 [14].

We have previously reported that elevated levels of circulating FFA in myocardial infarction [15] may contribute to the synthesis of inflammatory mediators through the activation of these receptors in immune, resident non-immune cells as well as in adipose tissue. Moreover, it has been recently suggested that TLR4, TLR2 and their signaling pathways participate in the inflammatory response caused by ischemia reperfusion injury [16].

MI causes the development of aseptic inflammatory acute-phase response, which in severe cases, leads to systemic inflammatory response syndrome [17]. Patients with MI, particularly with VO, demonstrated highly increased CRP levels. Increased CRP levels are observed in any tissue damage and aimed at the reorganization of necrotic myocardial cells and their residues which are highly toxic. Myocardial cells which are dead or damaged under ischemia and reperfusion secrete various endogenous molecules (alarmins) into the extracellular space. Alarmins are ligands of TLRs. Thus, the cells expressing TLRs with further synthesis of proinflammatory cytokines are activated.

In the case of myocardial infarction, obese patients with acute inflammatory responses include adipose tissue, which is already in a state of chronic inflammation and has a significant pro-inflammatory potential due to the macrophage polarization into M1 and a higher expression of TLRs in resident macrophages and adipocytes. However, these changes are more pronounced in VAT. Importantly, the expression of TLRs in circulating blood mononuclear cells is also higher in the presence of obesity. All of these changes ultimately lead to a severe inflammatory response in these patients. This is most obvious, but is not the only mechanism for increasing circulating pro-inflammatory cytokines in blood plasma of obese patients after MI, compared to lean patients.

According to the results of our study, there was insignificant a 1.4- and 1.9-fold increase in TNF-α and IL-1. TNF-α is a proinflammatory cytokine, primarily secreted from myeloid cells via activation of MAPK and NFκB signaling pathways, resulting in the release of other inflammatory cytokines, such as IL-1β and IL-6 [18]. The basic amount of TNF-α is synthesized resident macrophages [19]. Obesity is associated with elevated levels of TNF-α in plasma and adipose tissue, but excess weight loss leads to normalization of this parameter. However, the influence of TNF-α on immune response mostly results from its strengthen effect on the production of other interleukins, such as IL-6 and IL-1 β, rather than from a direct effect [18].

A series of in vitro experiments indicated that adipocytes produce IL-1β in obese people 2 times higher than in lean individuals. Neutralization of IL-1β and TNF-α in the culture medium significantly reduces the synthesis of IL-6 and IL-8 in adipocytes [20, 21]. Further clinical studies confirmed the data of in vitro experiments, indicating that the endogenous release of IL-1β and TNF-α from adipose tissue upregulates the synthesis of IL-6 and IL-8 [22].

According to our data, IL-6 levels increased 6.9-fold in patients with VO. Moreover, a relationship between IL-6 and VO in patients suffered from myocardial infarction may be associated with its synthesis not only by immune cells, but also by adipocytes. Similarly to TNF-α, WAT and plasma IL-6 expression correlate with increased body mass, waist circumference, and free fatty acid levels. IL-6 has been implicated as a marker for VO because VAT releases more IL-6 than SAT [20, 23].

The most significant changes were observed in IL-8 levels, which demonstrated a 24-fold increase. IL-8 belongs to the group of chemokines, providing chemotaxis and adhesion in the area of inflammation of various cell types (neutrophils, monocytes, T-cells, eosinophils and basophils). Monocytes, macrophages, lymphocytes, vascular endothelium, fibroblasts, epithelial cells may also produce IL-8. Its blood levels are elevated in people with obesity, correlating with body weight and TNF-α levels [24]. The main source of IL-8 in adipose tissue may be resident macrophages and adipocytes; thus, its synthesis is higher in visceral adipose tissue than in subcutaneous [24].

Unlike the above-mentioned cytokines, there are no data about possible synthesis of IL-12 in adipose tissue. Its increase is assumed to be associated with the presence of inflammation in the immediate area of myocardial damage. Accumulation of T cells and macrophages in atherosclerotic plaques and the formation of antibodies directed against plaque proteins suggests that adaptive immunity contributes to the development of atherosclerosis [25]. The results of clinical and experimental studies are consistent with this assumption. It has been established that IL-12 is an early inducer and a significant factor in the progression of atherosclerosis. Clinical data suggest using IL-12 blood concentration as a predictor of any adverse events after myocardial infarction within 1 year [26]. According to our data, IL-12 in patients with visceral obesity was also associated with the development of cardiovascular complication in the late post-MI period.

Apparently, intensification of inflammation in MI patients with VO is associated with a pronounced deficit of anti-inflammatory cytokine, IL-10, predominantly expressed by activated T lymphocytes (Th2-type). However, such cells as monocytes, macrophages, dendritic cells and B lymphocytes are involved in its synthesis as well. Lower levels of this cytokine have been found in the peripheral blood of patients with obesity and type 2 diabetes [23]. Suppressing the inflammatory response, IL-10 inhibits the production of IL-1α, IL-1β, TNF-α, IL-6, IL-8 and IL-12, primarily produced by activated monocytes, and reparative processes in myocardium, inhibition of fibrosis, enhancing vascularization. Furthermore, IL-10 may play a significant role in extracellular matrix remodeling by regulating expression of metalloproteinases and their inhibitors [23]. N.G. Frangogiannis et al. suggested that IL-10 may have a role in regulating extracellular matrix metabolism after experimental myocardial ischemia/reperfusion in dogs [26].

According to our data, a decrease in IL-10 levels has obvious adverse effect, particularly pronounced in the presence of VO. Deficiency of IL-10 was accompanied by the development of the imbalance of pro- and anti-inflammatory factors. IL-8/IL-10 ratio in patients with VO was 3.8 times higher than in lean individuals. In the presence of VO, despite clinical improvement in the post-MI period, adverse cardiovascular events closely related to IL-10 deficiency were registered.

In general, the dynamics of changes in cytokines levels indicates the need to improve the balance of pro- and anti-inflammatory cytokines by introducing new therapeutic approaches (cytokine inhibitors - receptor antibodies, suppression of cytokine synthesis by activated immune cells) regulating the activation process of inflammation. Physicians have much experience with the TNF blockers (infliximab and etanercept) in patients with psoriasis in preventing the development of cardiovascular events.

## Conclusion

Cytokine profile in MI patients with VO is characterized by an imbalance caused by elevated pro-inflammatory interleukins and decreased anti-inflammatory interleukins. An increase in the concentration of cytokines was as follows: a 1.3-fold increase in the levels of IL-1 and TNF-α, a 2-fold increase in IL-12 levels, a 6-fold increase in IL-6 levels and a 24-fold increase in IL-8 and CRP levels. Obesity in patients was associated with a marked increase in IL-6 and CRP levels. Dynamics of changes in the concentrations of IL-6, IL-12 and IL-10 is essential for the development of adverse cardiovascular events one year after MI. The obtained results suggest the use of immunomodulators to restore the balance of the pro- and anti-inflammatory cytokines in patients with VO. Drugs affecting the levels of IL-6, −12, −10, namely monoclonal antibody to IL-6 receptor, antibodies to IL-12, IL-10 inducers, seem to be promising in individuals with VO.

## Abbreviations

ATM: Adipose tissue macrophages
CAD: Coronary artery disease
CVD: Cardiovascular disease
DM: Diabetes mellitus
MI: Myocardial infarction
SAT: Subcutaneous adipose tissue
TLR: Toll-like receptors
VAT: Visceral adipose tissue
VO: Visceral obesity
WAT: White adipose tissue
